# Factors Affecting Technical Difficulty in Balloon Enteroscopy-Assisted Endoscopic Retrograde Cholangiopancreatography in Patients with Surgically Altered Anatomy

**DOI:** 10.3390/jcm10051100

**Published:** 2021-03-06

**Authors:** Naoya Izawa, Kohei Tsuchida, Keiichi Tominaga, Koh Fukushi, Fumi Sakuma, Ken Kashima, Yasuhito Kunogi, Mimari Kanazawa, Takanao Tanaka, Kazunori Nagashima, Takahito Minaguchi, Mari Iwasaki, Akira Yamamiya, Hidehito Jinnai, Akane Yamabe, Koki Hoshi, Takeshi Sugaya, Makoto Iijima, Kenichi Goda, Atsushi Irisawa

**Affiliations:** Department of Gastroenterology, Dokkyo Medical University, 880 Kitakobayashi, Mibu, Tochigi 321-0293, Japan; izawanao@dokkyomed.ac.jp (N.I.); tsuchida@dokkyomed.ac.jp (K.T.); d-fuku-k@dokkyomed.ac.jp (K.F.); sakuma-f@dokkyomed.ac.jp (F.S.); ken-k@dokkyomed.ac.jp (K.K.); ykunogi@dokkyomed.ac.jp (Y.K.); mimari77@dokkyomed.ac.jp (M.K.); tana1986@dokkyomed.ac.jp (T.T.); n-kazu@dokkyomed.ac.jp (K.N.); takahito@dokkyomed.ac.jp (T.M.); kizuki@dokkyomed.ac.jp (M.I.); akira-y@dokkyomed.ac.jp (A.Y.); h-jinnai@dokkyomed.ac.jp (H.J.); yamabe@dokkyomed.ac.jp (A.Y.); hoshi@dokkyomed.ac.jp (K.H.); t-sugaya@dokkyomed.ac.jp (T.S.); mkiijima@dokkyomed.ac.jp (M.I.); goda@dokkyomed.ac.jp (K.G.); irisawa@dokkyomed.ac.jp (A.I.)

**Keywords:** biliary intervention, balloon enteroscopy, endoscopic retrograde cholangiography, Roux-en-Y anastomosis

## Abstract

Success rates of balloon enteroscopy-assisted endoscopic retrograde cholangiopancreatography (BE-ERCP) for patients with a reconstructed intestinal tract after surgical procedures are unsatisfactory. We retrospectively investigated the factors associated with unsuccessful BE-ERCP. Ninety-one patients who had a reconstructed intestinal tract after gastrectomy or choledochojejunostomy were enrolled. Age, sex, operative method, malignancy, endoscope type, endoscopist’s skill, emergency procedure, and time required to reach the papilla/anastomosis were examined. The primary endpoints were the factors associated with unsuccessful BE-ERCP selective cannulation, while the secondary endpoints were the rate of reaching the papilla/anastomosis, causes of failure to reach the papilla/anastomosis, cannulation success rate, procedure success rate, and rate of adverse events. Younger age (odds ratio, 0.832; 95% CI, 0.706–0.982; *p* = 0.001) and Roux-en-Y partial gastrectomy (odds ratio, 54.9; 95% CI, 1.09–2763; *p* = 0.045) were associated with unsuccessful BE- ERCP. The rate of reaching the papilla/anastomosis was 92.3%, the success rate of biliary duct cannulation was 90.5%, procedure success rate was 78.0%, and the rate of adverse events was 5.6%. In conclusion, Roux-en-Y partial gastrectomy and younger age were associated with unsuccessful BE-ERCP. If BE-ERCP is extremely difficult to perform in such patients after Roux-en-Y partial gastrectomy, alternative procedures should be considered early.

## 1. Introduction

Since it was first reported in 1968 [1], endoscopic retrograde cholangiopancreatography (ERCP) has been the standard endoscopic procedure for diseases of the pancreaticobiliary tract. However, percutaneous or surgical treatments are frequently selected for such diseases in patients who have undergone gastrectomy or pancreatoduodenectomy (PD), because their reconstructed anatomies render endoscopy via the papillary approach difficult.

The successful use of balloon enteroscopy in ERCP for diagnosing and treating small-bowel diseases in a postoperatively reconstructed intestinal tract was first reported in 2005 [2]. Since then, balloon enteroscopy-assisted ERCP (BE-ERCP) has been widely used for patients with pancreatic or biliary disease with a reconstructed intestinal tract; however, success rates are unsatisfactory [3,4,5,6,7,8,9,10,11,12]. Fortunately, the recent development of a short-type balloon endoscope specialized for ERCP in patients with a postoperatively reconstructed intestinal tract has increased success rates [13,14,15,16,17,18,19,20,21,22,23,24,25,26]. However, the transpapillary approach is difficult to perform in some patients. In addition, excessive endoscopic manipulation in these cases can cause adverse events, such as intestinal perforation. If the difficulty in performing balloon enteroscopy can be predicted in advance, medical staff can prepare for alternative methods to ERCP, such as percutaneous bile duct drainage, surgery, and endoscopic ultrasound-guided bile duct drainage [27,28]. In this way, the endoscopic procedures performed in patients with reconstructed intestines would be safer and more accurate. In this retrospective study, we aimed to clarify the factors associated with unsuccessful BE-ERCP for pancreatic and biliary diseases in patients with postoperatively reconstructed intestines.

## 2. Materials and Methods

### 2.1. Study Design and Patient Population

Out of 2778 patients who underwent ERCP between April 2012 and January 2019 at the Department of Gastroenterology of Dokkyo Medical University Hospital, 91 had a postoperatively reconstructed intestinal tract and underwent their first BE-ERCP, and these subjects were included in this study. We retrospectively reviewed their patient records to analyze their age, sex, reconstruction method, primary disease (benign or malignant), balloon endoscope type (single or double), primary endoscopist’s skills (expert or trainee), emergency procedure (within 24 h of arrival at the hospital), time required to reach the papilla/anastomosis, success or failure of cannulation, success or failure of the procedure, and adverse events.

The primary endpoints were the factors associated with unsuccessful ERCP cannulation, and the secondary endpoints were the rate of reaching the papilla/anastomosis, causes of failure to reach the papilla/anastomosis, cannulation success rate, procedure success rate, and the rate of adverse events.

The bioethics committee of Dokkyo Medical University approved this study (Approval No. R-5-2, UMIN registration number UMIN000033963). We provided a means to withdraw from the study without omitting patients’ informed consent, assuring research subjects to be notified and our research information to be published on our website.

### 2.2. Definition of Sampling Items

In this study, we defined “successful selective cannulation” as the deep intubation of the imaging catheter into the biliary or pancreatic duct, “procedure success” as a successful diagnostic and therapeutic intervention, “successfully reaching the papilla or anastomosis” as reaching the papilla or anastomotic site, “reaching time” as the time between the start of oral intubation and the time point at which the cannulation site (papilla or choledochojejunostomy/pancreaticojejunostomy site) was reached, “expert” as a clinician who had performed ≥300 ERCPs, and “trainee” as a clinician who had performed <300 ERCPs.

The following four factors were examined as adverse events: post-ERCP pancreatitis, bleeding, gastrointestinal perforation, and infection. According to Cotton’s criteria, pancreatitis was classified as mild when the amylase level increased by threefold or more and the hospital stay lasted for 2–3 days, moderate when the hospital stay was extended to 4–10 days, and severe when the hospital stay was extended to >10 days [29]. With regard to bleeding, mild bleeding occurred when hemoglobin levels decreased by ≥2.0 g/dL within 24 h postoperatively with no transfusion requirement, moderate bleeding when ≤4 U of transfused blood was required, and severe bleeding when ≥5 U was required. Gastrointestinal perforations were assessed by enteroscopy, abdominal radiography, or computed tomography. Perforations that were resolved in ≤3 days were classified as mild, those requiring 4–10 days of treatment were moderate, and those requiring treatment and surgery for >10 days were severe. Furthermore, infections were classified as mild if a 38 °C fever decreased within 48 h, moderate if a high fever persisted and required ≥3 days of hospitalization, and severe if septic shock was noted.

### 2.3. Endoscopic Procedures

Comparing BE-ERCP and normal ERCP, the scope used in BE-ERCP has a working channel of 2.8–3.3 mm, which is thinner than the scope used in normal ERCP. In addition, since the treatment is performed under direct vision, the use of a sphincterotome is restricted. In addition, there are restrictions such as the inability to use a plastic stent with a diameter of 10 Fr.

All procedures were performed by one expert who had experience with ≥300 ERCPs and/or by several trainees who were skilled in performing colonoscopy and had experience with ≥100 ERCPs. If the primary endoscopist was a trainee and experienced difficulty in inserting the endoscope or cannulating the biliary or pancreatic duct, an expert took over as needed to continue the procedure.

Before starting the endoscopic procedure, all patients were administered pentazocine (15 mg) and midazolam (3–10 mg, depending on the patient’s status) for sedation and anesthesia. A short-type double-balloon endoscope (EI-530B; Fujifilm Medical Co., Ltd., Tokyo, Japan) or short-type single-balloon endoscope (SIF-H290S; Olympus Co., Tokyo, Japan) was fitted with a clear hood at the tip. When the insertion was difficult, abdominal compression was applied before attempting to insert again. An ERCP catheter (MTW Endoskopie, Wesel, Germany) and a guidewire (VisiGlide2; Olympus Co. or Jagwire; Boston Scientific Japan Co., Tokyo, Japan) were used. For insufflation, carbon dioxide was used.

### 2.4. Definitions of Adverse Events

Adverse events adopted the criteria defined by the American Society for Gastrointestinal Endoscopy (ASGE) [30,31]. An adverse event was one that prevents completion of the planned procedure (which does not include failure of completion because of technical failure, interference by poor preparation, disturbed anatomy, disease, or surgery) and/or results in hospital admission, prolongation of existing hospital stay, requiring another procedure (needing sedation/anesthesia), or subsequent medical consultation. Post-ERCP pancreatitis was defined as a case in which the serum amylase/lipase level was 3 times the upper limit of normal or higher.

Severity of adverse events were classified as follows: unplanned hospital admission or prolongation of hospital stay for 3 nights was considered mild: unplanned admission or prolongation for 4–10 nights was considered moderate: and unplanned admission or prolongation for 10 nights was considered severe. Surgery for an adverse event and permanent disability were classified as severe.

### 2.5. Statistical Analysis

Categorical and continuous variables were examined by Fisher’s exact test and the Mann–Whitney U test, respectively. Subsequently, two-factor multivariate logistic regression analysis was performed. For multivariate analysis, the factors and clinically significant variables that were indicated in univariate analysis were extracted, and *p* < 0.05 indicated statistical significance in multivariate analysis. All statistical data were analyzed by SPSS version 24 (IBM Corp., Armonk, NY, USA).

## 3. Results

### 3.1. Patient Characteristics

Ninety-one patients were assessed. The mean age was 72.6 years (standard deviation, 10.13 years), and 69 (75.8%) of the patients were male. Seventy-six patients underwent surgical operation for malignancy. The methods used for intestinal reconstruction were as follows: Roux-en-Y (R-Y) total gastrectomy (32 patients), R-Y partial gastrectomy (26 patients), Billroth II (B-II) reconstruction (8 patients), PD (18 patients), choledochojejunostomy in childhood (1 patient), Billroth I reconstruction and choledochojejunostomy (1 patient), and choledochojejunostomy (5 patients). Moreover, 58 patients had choledocholithiasis or intrahepatic stones, 24 had biliary strictures, 3 had anastomotic strictures, 3 had intraductal papillary mucinous neoplasms, 2 had pancreatic fistulas, and 1 had a pancreatic pseudocyst. Antithrombotic treatment had been performed for 18 (19.8%) cases. Single- and double-balloon endoscopes were used in 45 and 46 patients, respectively. The primary operator was an expert in 47 cases and a trainee in 44 cases. The procedure type using BE-ERCP was stenting in 54 and stone extraction in 14 patients. All patients who required treatment of papilla had undergone balloon dilation. The details are shown in Table 1.

### 3.2. Success of Reaching the Papilla/Anastomosis

The papilla or anastomosis was reached in 92.3% (84/91) of patients. The intubation rates by surgical reconstructive procedure were as follows: R-Y total gastrectomy, 96.8% (30/31); R-Y partial gastrectomy, 92.3% (24/26); B-II, 87.5% (7/8); PD, 88.8% (16/18); and others, 83.3% (5/6) (Table 2). However, the ampulla/biliary anastomosis could not be reached in some patients because of malignant strictures (three patients), long Y limb (two patients), and long procedure duration (two patients), and was subsequently treated conservatively (one patient), by percutaneous transhepatic biliary drainage (PTBD) (two patients), or by repeat ERCP (three patients). In univariate analysis, factors such as age, sex, reconstruction method, normal stomach or gastrectomy, primary disease (benign or malignant), endoscope type (single or double balloon), primary endoscopist (expert or trainee), and emergency procedure were not associated with failure to reach the papilla or anastomosis with the balloon endoscope (Supplementary Table S1).

### 3.3. Selective Cannulation

Selective cannulation was successful in 90.5% (76/84) of patients whose papilla/anastomosis was reached, and the cannulation success rates by surgical procedure were as follows: R-Y total gastrectomy, 96.8% (30/31); R-Y partial gastrectomy, 75% (18/24); B-II, 100% (7/7); PD, 100% (16/16); and others, 83.3% (5/6) (Table 2).

Difficult cannulation was caused by tumor infiltration in three and four cases in which the infiltration and adhesion could not be seen in front of the papilla, respectively. Patients who did not undergo cannulation were treated conservatively (one patient), by PTBD (one patient), repeat ERCP (one patient), ERCP with the rendezvous technique (three patients), or by surgery (one patient). Through univariate analysis, the association between the following 10 factors and unsuccessful selective cannulation of the biliary/pancreatic duct was analyzed: age, sex, reconstructive procedure, normal stomach or gastrectomy, the native papilla or anastomosis, primary disease (benign or malignant), endoscope type (single- or double balloon), primary endoscopist (expert or trainee), emergency procedure, and reaching time. Our results suggest that age (*p* = 0.01), the reconstructive procedure (*p* = 0.034), malignant disease (*p* = 0.039), endoscope type (*p* = 0.166), skill level (*p* = 0.184), and reaching time (*p* = 0.133) were associated with unsuccessful selective cannulation (Table 3). In multivariate analysis using the six factors identified in univariate analysis, age (odds ratio (OR), 0.832; 95% confidence interval (CI), 0.706–0.982; *p* = 0.001) and R-Y partial gastrectomy (OR, 54.9; 95% CI, 1.09–2763; *p* = 0.045) were independently associated with unsuccessful selective cannulation (Table 4).

### 3.4. Procedure Success

Although selective cannulation was successful in 76 patients whose papilla/anastomosis was reached, the whole procedure was successful in only 71 (78.0%) patients because five were unable to undergo treatment despite achieving cannulation. The clinical success rates by surgical procedure were as follows: R-Y total gastrectomy, 87.1% (27/31); R-Y partial gastrectomy, 65.4% (17/26); B-II, 87.5% (7/8); PD, 83.3% (15/18); and others, 83.3% (5/6) (Table 2). In univariate analysis, factors such as age, sex, reconstructive procedure, normal stomach or gastrectomy, the native papilla or anastomosis, primary disease (benign or malignant), endoscope type (single or double balloon), primary endoscopist (expert or trainee), and emergency procedure were not associated with failure of the whole procedure (Supplementary Table S2).

### 3.5. Adverse Events

Of the 91 patients, five (5.6%) experienced adverse events. Four patients developed moderate postoperative acute pancreatitis, and one developed mild intra-abdominal perforation (Table 5). All adverse events improved with conservative therapy, and no serious adverse events occurred. The intestinal perforation was caused by traumatic guidewire insertion, and its symptoms were mild and improved by fasting, fluid replacement, and antibiotic administration. Patients who underwent metallic stent placement (*n* = 1), biliary drainage (*n* = 3), and stone removal surgery (*n* = 1) experienced complications.

## 4. Discussion

Haruta et al. [2] first reported the use of ERCP in patients with a reconstructed intestinal tract. Currently, this technique is recognized as an effective drainage method for patients with biliary tract disease with a reconstructed intestinal tract. The success and complication rates of BE-ERCP in patients with a reconstructed intestinal tract are reportedly 63–95% and 0.0–12.4%, respectively [4,5,6,7,8,9,10,11,12,13,14,15,16,17,18,19,20,21,22,23]. In our study, the cannulation success rate in patients whose papilla/anastomosis was reached was 90.5%, which is similar to the rates in previous reports of this procedure using short-type balloon endoscopes (89–100%) [13,14,15,16,17,18,19,20,21,22,23,24]. Success rates have greatly increased, owing to the development of new features in balloon enteroscopes, such as passive bending, and new devices specialized for BE-ERCP [23,24,25,26]. However, the outcomes of BE-ERCP in patients with biliary and pancreatic diseases with a reconstructed intestinal tract remain less satisfactory than in those patients without such health conditions. This difference is caused by the use of various surgical methods, the individual differences in the length of reconstructed intestinal tracts despite performance of the same surgical procedure, and the presence of adhesions left by the surgical procedures.

The reported factors associated with cannulation failure in BE-ERCP in previous literature are shown in the Table 6 [12,23,32,33]. Thus, various significant risk factors for BE-ERCP have been reported. Our study showed that younger age and R-Y partial gastrostomy were the risk factors for cannulation failure in patients whose papilla/anastomosis was successfully reached. In contrast, Yane et al. [23] reported that age is not a factor of cannulation failure in short-type single-balloon enteroscopy. Unfortunately, the reason why younger age might be a risk factor for unsuccessful BE-ERCP remains unclear. However, adhesions may be a possible reason. Liu et al. [12] reported that patients who had undergone childhood surgery such as the Kasai procedure for biliary atresia had the lowest success rate, and speculated that adhesion was more likely to occur because the postoperative course is generally longer in young people. Our patients might have had strong adhesions. Hence, this issue should be examined with more cases. Regarding the intestinal tract reconstruction method, Skinner et al. [34] reported that the procedure was easiest with B-II reconstruction and most difficult with R-Y reconstruction. Thus, we assumed that the procedure was particularly difficult in R-Y reconstruction with partial gastrectomy because the endoscope can bend easily in the gastric remnant, making the endoscope difficult to support; this notion might explain why cannulation becomes difficult. In addition, Kawaguchi et al. [35] analyzed the factors contributing to failure to reach the blind end in patients with intestinal tract reconstruction, and they demonstrated that R-Y without gastrectomy (*p* = 0.001; OR, 5.73) is a significant factor for procedure failure. These reports support our results. Incidentally, we did not clarify the significant factors associated with whole-procedure failure.

In cases of unsuccessful endoscopic drainage, PTBD and surgery are the preferred alternatives to ERCP. PTBD, which has been widely performed, has a reported technical success rate of 95% and complication rate of 5.7–12% [36,37,38,39,40]; these rates are approximately equal to those of ERCP using balloon enteroscopy on reconstructed intestinal tracts. Recently, endoscopic ultrasound-guided biliary drainage (EUS-BD) was performed for unsuccessful ERCP [41], attaining a success rate similar to that of PTBD (technical success rate, 77–94%) and a complication rate of 19–27% [27,28]. EUS-BD can also be indicated for patients with a reconstructed intestinal tract and is actively used for cases of unsuccessful ERCP using balloon enteroscopy. Anterograde drainage is considered most physiologically optimal, especially considering its minimal invasiveness. Our study showed that R-Y partial gastrectomy (OR, 54.9; *p* = 0.045) is associated with unsuccessful ERCP.

Although BE-ERCP (papillary or transbiliary/pancreaticojejunostomic drainage) is the first-line management for patients with a reconstructed intestinal tract, PTBD or EUS-BD may be applied instead when BE-ERCP is extremely difficult in the case of R-Y partial gastrectomy [42,43]. These alternative treatments may also be considered in younger patients. However, the efficacy and safety of these alternative treatments remain unknown for younger patients. Therefore, these treatment methods should be considered according to the condition/status of the patient.

In ERCP, the incidence of adverse events is higher in patients with a postoperative reconstructed intestinal tract than in those with unaltered anatomies [44]. The overall incidence of adverse events reportedly ranges from 0.0 to 12.4% [4,5,6,7,8,9,10,11,12,13,14,15,16,17,18,19,20,21,22,23,24], which was similarly observed in our findings. No severe complications occurred among the patients in this study. However, although rare, adverse events such as perforations and bleeding may occur in patients with severe adhesion [32,45,46,47]. In these patients, factors that inhibit endoscope insertion (e.g., severe adhesion) should be expected, and switching to an alternative therapy might be considered early in the treatment course to prevent unnecessary adverse events.

The main limitations of this study are as follows: it is a retrospective study, and it was conducted in a single institution. Moreover, our study showed that younger age was a predictor of difficulty in performing BE-ERCP, while the skill/experience of the endoscopist was not a risk factor. These results were statistically evaluated in only eight patients who had failed cannulation in BE-ERCP, thus, this is likely to be the effect of uncontrollable bias. Nevertheless, no other studies on this topic have included the reaching time or reconstructive method as factors; thus, our results are valuable for improving future treatments.

## 5. Conclusions

The factors associated with unsuccessful balloon BE-ERCP in patients with a reconstructed intestinal tract were R-Y partial gastrectomy and younger age. When performing BE-ERCP is extremely difficult in patients with reconstructed intestinal tracts after R-Y partial gastrectomy, alternative procedures such as PTBD or EUS-BD may be considered early.

## Figures and Tables

**Table 1:** Characteristics of patients who underwent endoscopic retrograde cholangiopancreatography (ERCP).

Patients, *n*	91
Age, years, mean (SD)	72.66 (10.132)
Sex, male, *n* (%)	69 (75.8)
Objective diseases (malignant/benign)	76:15
Reconstructive procedure, *n* (%)	
Roux-en-Y total gastrectomy	32 (35.2)
Roux-en-Y partial gastrectomy	26 (28.6)
Billroth II gastrectomy	8 (8.8)
Pancreaticoduodenectomy	18 (19.8)
Others	7 (7.7)
Diagnosis, *n* (%)	
Bile duct stone	58 (63.7)
Bile duct stricture	24 (26.4)
Stricture of choledo- or hepaticojejunal anastomosis	3 (3.3)
Others	6 (6.6)
Talking antithrombotic agent, *n* (%)	18 (19.8)
Antiplatelet, *n* (%)	10 (11.0)
Anticoagulant, *n* (%)	10 (11.0)
Emergency procedure, *n* (%)	41 (45.1)
Endoscope type, single, *n* (%)	45 (49.5)
Skill, expert, *n* (%)	47 (51.6)
Kinds of procedure	
EBD (plastic stent), *n* (%)	49 (53.8)
EBD (metal stent), *n* (%)	3 (3.3)
ENBD, *n* (%)	2 (2.2)
Stone extraction, *n* (%)	14 (15.4)
Others, *n* (%)	23 (25.3)

Abbreviations: EBD: endoscopic bile drainage, ENBD: endoscopic nasal bile drainage.

**Table 2:** Associations of anatomy with success rate of enteroscopy, cannulation, and procedure.

	Reaching the Scope to the Ampulla/Biliary Anastomosis	Selective Cannulation	Procedure Success
Rate	92.3% (84/91)	90.5% (76/84)	78.0% (71/91)
Reconstructive procedure			
Roux-en-Y total gastrectomy	96.9% (31/32)	96.8% (30/31)	87.1% (27/31)
Roux-en-Y gastrectomy	92.3% (24/26)	75.0% (18/24)	65.4% (17/26)
Billroth II	87.5% (7/8)	100% (7/7)	87.5% (7/8)
Pancreaticoduodenectomy	88.9% (16/18)	100% (16/16)	83.3% (15/18)
Others	85.7% (6/7)	83.3% (5/6)	71.4% (5/7)

**Table 3:** Association of each factor with success and failure in patients undergoing cannulation.

	Success	Failure	*p* Value
Cannulation, no. of cases	76	8	
Age, mean (SD)	74.1 (9.66)	65.5 (7.09)	0.01
Sex, male/female, (*n*)	58/18	6/2	0.613
Reconstructive procedure, R-Y total gastrectomy/R-Y partial gastrectomy/B-II/PD/others, (*n*)	30/18/7/16/5	1/6/0/0/1	0.034
Gastrectomy, yes/no, (*n*)	71/5	8/0	0.598
Papilla, native papilla/anastomosis, (*n*)	58/17	7/1	0.446
Malignant disease, yes/no, (*n*)	12/64	4/4	0.039
Endoscope type, single/double, (*n*)	38/38	2/6	0.166
Skill, expert/trainee, (*n*)	39/37	6/2	0.184
Emergency, emergency procedure/secondary Procedure, (*n*)	36/40	4/4	0.588
Reaching time, mean (SD)	33.7 (20.9)	48.4 (29.0)	0.133

Abbreviations: B-II, Billroth II; PD, pancreaticoduodenectomy; R-Y, Roux-en-Y.

**Table 5:** Adverse events in patients undergoing ERCP (*n* = 91).

	*n* (%)	Severity Grade
No adverse events	86 (94.5)	
Total adverse events	5 (5.5)	Mild: 1, Moderate: 4
Pancreatitis	4 (4.4)	Moderate: 4
Intestinal perforation	1 (1.1)	Mild: 1

**Table 6:** Outcomes of previous reports on the association between BE-ERCP and related clinical factors.

Authors (Year)	No. of Patients	Endoscope Used for Cannulation	Failure Factor of Cannulation
Liu K et al. (2017) [12]	52	DBE	patients with surgically corrected biliary atresia, post-transplant patients with second operation
Yane K et al. (2017) [23]	117	short SBE	indication for pancreatic disease, first ERCP attempt, no transparent hood
Uchida D et al. (2020) [32]	319	short DBE	Roux-en-Y reconstruction, first-time short DBE-ERCP
Tanisaka Y et al. (2019) [33]	121	short SBE	malignant biliary obstruction, first ERCP attempt, Roux-en-Y reconstruction.

Abbreviations: BE-ERCP: balloon enteroscopy-assisted endoscopic retrograde cholangiopancreatography, SBE: single balloon enteroscope, DBE: double balloon enteroscope.

**Table 4:** Association between cannulation in ERCP and related clinic factors with a logistic regression model (*n* = 84).

	OR	95% CI	*p* Value
Age	0.832	0.706–0.982	0.001
Reconstructive procedure			
R-Y total gastrectomy	1		
R-Y partial gastrectomy	54.9	1.09–2763	0.045
B-II	n/a		
PD	n/a		
Others	2.16	0.075–62.176	0.53
Malignant disease	3.29	0.199–54.443	0.406
Trainee	0.185	0.012–2.868	0.228
Single-balloon enteroscopy	0.885	0.062–2.649	0.928
Reaching time	1.041	0.996–1.089	0.076

Abbreviations: B-II, Billroth II; CI, confidence interval; n/a, not applicable; OR, odds ratio; PD, pancreaticoduodenectomy; R-Y, Roux-en-Y.

## Data Availability

No new data were created or analyzed in this study. Data sharing is not applicable to this article.

## Supplementary Materials

The following are available online at https://www.mdpi.com/2077-0383/10/5/1100/s1, Supplementary Table S1: Association of each factor with success and failure in patients undergoing enteroscopy. Supplementary Table S2: Association of each factor with success and failure in patients undergoing ERCP procedure.
