# Supplementary material for: Factors Affecting Technical Difficulty in Balloon Enteroscopy-Assisted Endoscopic Retrograde Cholangiopancreatography in Patients with Surgically Altered Anatomy
Source: J Clin Med. 2021 Mar 6;10(5):1100. doi: 10.3390/jcm10051100 (PMC7961549; doi:10.3390/jcm10051100)
Supplement: Supplementary file 1 [file jcm-10-01100-s001.pdf]

Supplementary Table S1. Association of each factor with success and failure in patients undergoing enteroscopy.

	**Success**	**Failure**
Enteroscopy, No. of cases	*84*	*7*
Age, mean (SD)	73.2 (9.75)	65.4 (12.63)
Sex, n, male/female	64/20	5/2
Anatomy, n, R-Y total gastrectomy/R-Y partial gastrectomy/B-II/PD/others	31/24/7/16/6	1/2/1/2/1
Gastrectomy n, yes/no	79/5	6/1
Malignant disease, n, yes/no	16/68	2/5
Endoscope type, n, single/double	40/44	5/2
Skill, n, expert/ trainee	45/39	2/5
Emergency, n, emergency procedure/secondary procedure	40/44	1/6

Abbreviations: B-II, Billroth II; PD, Pancreaticoduodenectomy; R-Y, Roux-en-Y.

Supplementary Table S2. Association of each factor with success and failure in patients undergoing ERCP procedure.

	**Success**	**Failure**	***p* value**
Procedure, No. of cases	*71*	*20*	
Age, mean (SD)	74.9 (8.965)	64.7 (10.179)	0.833
Sex, n, male/female	55/16	6/14	0.339
Anatomy, n, R-Y total gastrectomy/R-Y partial gastrectomy/B-II/PD/others	27/17/7/15/5	4/9/0/1/2	0.077
gastrectomy n, yes/no	66/5	19/1	0.606
Papilla n, native papilla/anastomosis	54/16	17/3	0.338
Malignant disease, n, yes/no	12/59	6/14	0.162
Endoscope type, n, single/double	35/36	10/10	0.578
Skill, n, expert/trainee	38/33	9/11	0.337
Emergency, n, emergency procedure/secondary procedure	34/37	7/13	0.222

Abbreviations: B-II, Billroth II; PD, Pancreaticoduodenectomy; R-Y, Roux-en-Y.
